# Planar Cell Polarity and E-Cadherin in Tissue-Scale Shape Changes in *Drosophila* Embryos

**DOI:** 10.3389/fcell.2020.619958

**Published:** 2020-12-23

**Authors:** Deqing Kong, Jörg Großhans

**Affiliations:** Department of Biology, Philipps-University Marburg, Marburg, Germany

**Keywords:** *Drosophila* embryonic epithelium, DE-cadherin, planar polarity, non-muscle myosin-II, tissue-scale shape changes

## Abstract

Planar cell polarity and anisotropic cell behavior play critical roles in large-scale epithelial morphogenesis, homeostasis, wound repair, and regeneration. Cell–Cell communication and mechano-transduction in the second to minute scale mediated by E-cadherin complexes play a central role in the coordination and self-organization of cellular activities, such as junction dynamics, cell shape changes, and cell rearrangement. Here we review the current understanding in the interplay of cell polarity and cell dynamics during body axis elongation and dorsal closure in *Drosophila* embryos with a focus on E-cadherin dynamics in linking cell and tissue polarization and tissue-scale shape changes.

## Introduction

Epithelia constitute the surface of organs in multicellular organisms and the units of many morphogenetic processes. Epithelial cells adhere to one another to form two-dimensional sheets and constitute permeability barriers for compartmentalization of the body, which is essential for the physiology and protection of the organs and even the whole organisms. Despite their physical integrity and stability, epithelial sheets are intrinsically dynamic and able to restructure in a time scale as fast as minutes (Gumbiner, 1992; Leptin, 1994; Lye and Sanson, 2011; Lv et al., 2019). During morphogenesis, epithelia undergo tissue-scale morphology changes, such as extension, closure, invagination, tubulation, and wrapping. Underlying those morphogenetic processes are cellular activities such as junction remodeling, cell shape changes, and cell rearrangement.

Planar polarity is based on molecular asymmetries within the epithelial sheet and cells and impinges on the cellular activities leading to tissue-scale shape changes. Cell junctions are at the center of the transition from cells to tissue. The mechanical link between is constituted by adherens junctions with E-cadherin (E-cad)–catenin complexes as the central component. Together with numerous associated proteins varying between cell types and developmental stages, the E-cad complex provides a mechanical link between the actomyosin networks of adjacent cells and coordinates their activities via mechanotransduction (Maître and Heisenberg, 2013; Leckband and de Rooij, 2014; Charras and Yap, 2018).

In this review, we will focus on recent progress in two processes of *Drosophila* embryogenesis, i.e., germband extension and dorsal closure. With these two case studies, we will discuss how cell and tissue polarization are coordinated to give rise to tissue-scale changes in visible morphology.

## *Drosophila* Embryonic Epithelium

The first epitheliogenesis, termed cellularization, in *Drosophila* development is initiated when the zygotic genome is activated at the transition from syncytial to cellular morphology (Schmidt and Grosshans, 2018). Cell polarization and epithelial sheet formation are intrinsically linked during cellularization. As the plasma membrane ingresses, the cell cortex becomes polarized as visible by segregation of cortical markers. Initially assembling into spot junctions distributed along the lateral furrow, the E-cad–catenin complex coalesces into unmatured adherens junctions at the typical subapical position only by the end of cellularization. During gastrulation, the epithelial epidermis undergoes stage and position-dependent morphogenetic movements, such as tissue invagination (Leptin, 2005; Martin, 2020), folding (Wang et al., 2012), convergent extension (Kong et al., 2017; Paré and Zallen, 2020), compartmental boundaries formation (Sharrock and Sanson, 2020), and dorsal closure (Hayes and Solon, 2017; Kiehart et al., 2017) to name the most prominent ones.

## DE-Cadherin and Adherens Junctions

**FIGURE 1:**
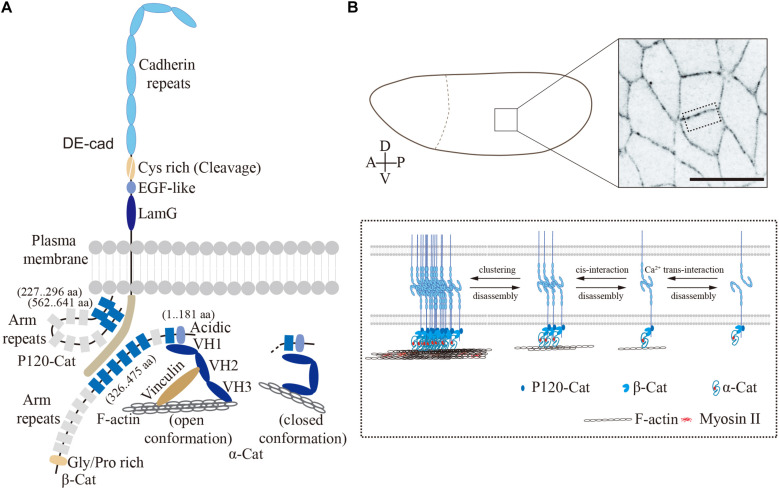
*Drosophila* embryonic epithelium. **(A)** The cadherin–catenin complex in *Drosophila* (modified from Harris, 2012). The single-transmembrane protein, DE-cadherin mediates cell–cell adhesion through homophilic interactions between its extracellular domain. In contrast, its cytoplasmic domain binding with p120-catenin, β-catenin, and α-catenin as cadherin–catenin complex forms the core of adherens junctions. Protein domains and interacting regions are indicated. **(B)** Confocal image of lateral epithelium from an embryo expressing E-Cad-GFP, scale bar is 10 μm. The close-up view on the bottom shows the adherens junctions and DE-cad clusters in *Drosophila* epithelium. DE-cad forms the adherens junctions via homophilic binding of N-terminal-most four extracellular cadherin domains, while the cytoplasmic domain binding with actomyosin network via Arm/ß-catenin and α-catenin.

*Drosophila* E-cadherin (DE-cadherin, DE-cad), known as Shotgun (Shg) in *Drosophila*, was identified as Armadillo (β-catenin) associated glycoprotein (Oda et al., 1994) and by the zygotic lethal mutation *shotgun* (Tepass et al., 1996; Uemura et al., 1996). Similar to classical cadherins in vertebrates, DE-cad is a single-transmembrane protein with seven cadherin repeats at its extracellular N-terminal region, followed by a cysteine-rich region, an EGF-like region and a laminin G domain. The cytoplasmic part contains binding sites for p120-catenin (Myster et al., 2003) and β-catenin (Pai et al., 1996), which leads to the assembly of the stereotypic cadherin–catenin complex at the core of adherens junctions (Figure 1A). DE-cad is proteolytically cleaved at its cysteine-rich region into two fragments after translation. The two fragments remain associated, however, via non-covalent interactions to form the mature protein (Oda and Tsukita, 1999). E-cad molecules undergo stable Ca^2+^-dependent homotypic interactions in trans between adjacent cells (Oda et al., 1994). The mammalian E-cad contains five cadherin repeats at its N-terminal portion. Of these, the most N-terminal-most cadherin domain engages in homophilic binding. In *Drosophila*, the four N-terminal-most cadherin domains have been reported to mediate the trans interaction (Figure 1B) (Nishiguchi et al., 2016). Beside the polypeptide backbone, post-translational modifications, such as glycosylation and phosphorylation, are essential for the functions of DE-cad and epithelial morphogenesis (Zhang et al., 2014; Chen et al., 2017).

In the fertilized egg and syncytial stage, DE-cad is more or less uniformly distributed within the plasma membrane and intracellular vesicles. The first junctions involving DE-cad are observed during cellularization (Cox et al., 1996; Müller and Wieschaus, 1996). Generic adherens junctions at a subapical position with an F-actin belt form and mature during late cellularization and gastrulation (stage 7–9), when the DE-cad density increases and coalesces into clusters and stable microdomains (Harris and Peifer, 2004; Cavey et al., 2008; Truong Quang et al., 2013). Beside the Ca^2+^-dependent interactions in trans, E-cadherin molecules bind to each other in cis within the same lipid bilayer to form super-molecular clusters (Figure 1B). Similar to mammalian cells (Engl et al., 2014; Wu et al., 2015), the DE-cad clusters require interactions with F-actin (Truong Quang et al., 2013). Non-muscle Myosin-II (Myosin-II) dependent tensile forces promote DE-cad clustering at cell contacts (Kale et al., 2018). However, the detailed mechanisms by which the cell cortex impinges on the DE-cad clusters remain elusive. *In vitro* studies revealed a function of the intracellular cadherin–catenin complex as a force sensor. Mechanical forces from actin cytoskeleton induce long-lived bonds in the cadherin-catenin complex (Buckley et al., 2014) and promote binding of the actin-binding protein Vinculin to α-catenin. In this way, a self-reinforcing system is established to strengthen the linkage between E-cad clusters and the actin cytoskeleton.

Armadillo is the *Drosophila* homolog of β-catenin, whose 13 copies of so-called Armadillo repeats are its characteristic feature (Peifer and Wieschaus, 1990). The N-terminal region and the first Armadillo repeat bind to α-catenin, while Armadillo repeats 3–8 are necessary and sufficient for DE-cad binding (Orsulic and Peifer, 1996; Pai et al., 1996), thus generating a bridge between the plasma membrane with E-cad and α-catenin with F-actin.

Within α-catenin, the VH1 domain mediates the interaction with ß-catenin (Oda et al., 1993; Pai et al., 1996) and the VH3 domain, binding to F-actin (Pokutta et al., 2008). Vertebrate α-catenin undergoes a reversible force-dependent change between two stable conformations (Choi et al., 2012; Rangarajan and Izard, 2012; Yao et al., 2014; Charras and Yap, 2018; Ishiyama et al., 2018). In the open conformation, when force is applied, α-catenin is bound on the one side to the Cadherin complex and the other side via the central mechanosensitive modulatory (M) domain to the D1 domain of Vinculin, thus bridging adherens junctions and F-actin. In contrast, when no force is applied, α-catenin changes into closed conformation with an inaccessible M-domain. In the closed conformation, α-catenin binds only to the Cadherin complex but not to Vinculin and its associated F-actin (Figure 1A). In *Drosophila* embryos, Vinculin colocalizes with E-cad (Kale et al., 2018), which is promoted by intracellular contracting forces and reduced following tissue relaxation (Kong et al., 2019).

p120-catenin is involved in endocytosis of the dynamic E-cadherin and Bazooka complexes in *Drosophila* embryos (Bulgakova and Brown, 2016). Binding of p120-catenin also appears to be mechanosensitive as recent research from *Drosophila* wing epithelium. In this system, p120-catenin is involved in E-cadherin turnover and epithelial viscoelasticity (Iyer et al., 2019). The numerous proteins associated with adherens junctions beyond the core complex have been discussed and reviewed by Harris (2012), for example.

In summary, adherens junctions with the E-cad–catenin complex at its core link the actin cytoskeletons of two neighboring cells in an epithelium (Figure 1). Spatial and temporal modulation of the complexes is a central feature of dynamic epithelia during embryogenesis.

## Germband Extension: From Anterior–Posterior Pattern to Planar Polarity to Cell Intercalation

**FIGURE 2:**
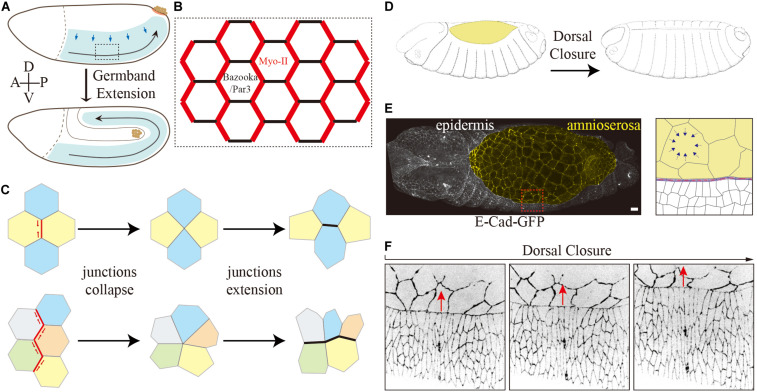
Two tissue-scale shapes change events during *Drosophila* embryogenesis. **(A–C)** Germband extension. **(A)** Schematic drawing of germband extension. **(B)** Polarized planer polarity of the lateral epidermis during germband extension. Myosin-II is enriched explicitly at AP interfaces, and Bazooka/PAR-3 is enriched at DV interfaces conversely in the intercalating cells. **(C)** Schematic representation of a simple and rosette-type T1 transition. **(D–F)** Dorsal closure. Schematic drawing of dorsal closure. Images modified from Hartenstein (1993) **(D)**. It involves two different types of epithelial tissues and their coordination: amnioserosa (yellow) and epidermal cells **(E)**. Confocal image of an embryo expressing E-Cad-GFP. The magnified view on the right shows the interface of amnioserosa and epidermal cells. The blue arrows indicate cell contraction. The leading edge cells polarize by the accumulation of filamentous actin and myosin II at the epidermis–amnioserosa interface in the form of an F-actin cable. **(F)** Time series of an embryo expressing E-Cad-GFP shows the shape of surrounding epidermal cells elongates along the dorsal–ventral axis during dorsal closure. The red arrows indicate the movements of epidermal cells during dorsal closure.

*Drosophila* germband extension serves as a paradigm for axis elongation by convergence and extension of an epithelial sheet (Figures 2A–C) (Kong et al., 2017). During germband extension, the lateral epidermis increases its length more than two-fold along the anterior–posterior (AP) axis, while correspondingly narrowing along the dorsal–ventral (DV) axis. The elongation of the tissue is largely due to polarized cell rearrangement by neighbor exchanges (Figures 2A–C) (Irvine and Wieschaus, 1994), whose key process is junction remodeling similar to a topological T1 transition (Figure 2C) (Weaire and Rivier, 1984). T1 transitions consist of two phases: (1) collapse of a junction (DV orientation, AP interfaces) leading to fusion of two 3x vertices into a single 4x vertex and (2) expansion of a new junction in perpendicular orientation (AP direction, DV interfaces) creating two new 3x vertices out of the transient 4x vertex (Figure 2C) (Bertet et al., 2004). A complex variant of T1 transitions, rosettes, are observed later in germband extension when multiple junctions collapse simultaneously to generate multiple fold vertices (rosette), which subsequently resolved by the formation of multiple new junctions (Figure 2C) (Blankenship et al., 2006).

Myosin-II and the junction-associated actomyosin network on the one side and Baz/PAR-3 and adherens junction proteins on the other side show a complementary and polarized distribution at the junctions and thus reflect a planar polarity (Figure 2B) (Bertet et al., 2004; Zallen and Wieschaus, 2004; Blankenship et al., 2006). Myosin-II and F-actin, enriched at AP interfaces, generate contractile forces leading to junction collapse (Bertet et al., 2004; Zallen and Wieschaus, 2004; Blankenship et al., 2006; Rauzi et al., 2008; Fernandez-Gonzalez et al., 2009). The force is probably generated by a flow of contractile filaments away from the adherens junctions at the apical cortex (medial). In an isotropic case, this leads to apical contractions (Martin et al., 2009; Kong et al., 2019). In the planar polarized situation of the lateral epidermis, the force acts in an anisotropic fashion mainly on the junctions with a DV orientation to induce a junction collapse (Rauzi et al., 2010).

The cortical and junctional actomyosin network is the force-generating machinery in the cell. Myosin-II exists as an inactive hexametric complex, consisting of two heavy chains, two essential light chains (ELC) and two regulatory light chains (RLC) (Hartman and Spudich, 2012). The Rho signaling pathway is essential for this polarization in the lateral epidermis and Myosin-II activity. Myosin-II is activated by phosphorylation of the RLCs by Rho-kinase (Rok) among other protein kinases. During germband extension, Rok is enriched at AP interface (de Matos Simões et al., 2010), and activated by the G protein-coupled receptor (GPRC)-Rho1 signaling (Kerridge et al., 2016), involving Dp114RhoGEF and the subunits of trimeric G proteins, Gβ13F/Gγ1 (De Las Bayonas et al., 2019). The asymmetry in Rho1 and Rok activation leads to polarized Myosin-II activation at AP junctions (de Matos Simões et al., 2010; Simões et al., 2014). Ligands of the FGF family control the assembly of rosette-like mechanosensory organs in the migrating lateral line primordium of the zebrafish (Lecaudey et al., 2008; Nechiporuk and Raible, 2008). It was revealed that Fgfr-Ras-MAPK signaling is required for apical constriction via apical positioning of Rho-associated kinase (Harding and Nechiporuk, 2012), which could be a potential further mechanism for acto-myosin activation during *Drosophila* germband extension. In parallel Rho1 also activates the formin Diaphanous (Dia), which initiates DE-cad endocytosis leading to depletion of α-catenin (Levayer et al., 2011) and Baz/PAR-3 at AP interface (de Matos Simões et al., 2010; Simões et al., 2014).

The initial signal for polarization is provided by the striped expression of anteroposterior patterning genes (Irvine and Wieschaus, 1994). The striped and staggered expression of the primary pair-rule genes, *runt*, *eve*, and *paired* imposes a planar polarity on the tissue, which guides the orientation of T1 transitions and thus the directionality of cell intercalation. AP patterning of *Drosophila* embryo is controlled by a hierarchical genetic cascade starting with localized maternal determinants to the zygotic gap, pair-rule, and segment polarity genes (Nasiadka et al., 2002). The link between patterning genes and planar cell polarity is mediated by members of the Toll receptor (protein) family (Paré et al., 2014). The staggered expression of primary pair-rule genes induces a corresponding stripe-like expression of Toll-2, 6, 8 (Eldon et al., 1994; Kambris et al., 2002). Heterophilic interfaces at the AP interfaces between these Toll-2,6,8 proteins, lacking at DV interfaces, induce specific signaling different between AP and DV interfaces (Paré et al., 2014; Tetley et al., 2016). The molecular link between Toll receptors and Myo-II may be provided by the adhesion GPCR Cirl, which can bind to Toll-8 (Lavalou et al., 2020). The Toll-8-Cirl complex self-organizes to generate local asymmetric interfaces which are essential for planar polarizations of contractile interfaces. In addition to Toll-Rho signaling, the classical planar polarity system involving Frizzled and which mediates planar polarity in wings and eye imaginal discs may also be involved in germband extension (Yang and Mlodzik, 2015). Although Frizzled was reported to be enriched on vertical junctions during cell intercalation (Warrington et al., 2013), neither the Frizzled nor the major Wnt effector Disheveled appears to be required for germ-band extension (Zallen and Wieschaus, 2004; Warrington et al., 2013).

## Dorsal Closure

Dorsal closure is another prominent morphogenetic process in *Drosophila* embryogenesis (Figures 2D–F) (Hayes and Solon, 2017; Kiehart et al., 2017). Dorsal closure involves two types of epithelial tissues and their coordination, i.e., the squamous amnioserosa and the columnar dorsal–lateral epidermis. After germband retraction, the extraembryonic amnioserosa bridges the left and right sheets of the dorsal epidermis (Figure 2E). Within about 4 h, the two lateral epidermal sheets on both sides of the embryo move toward the dorsal midline while the amnioserosa retreats and finally disappears (Figure 2D). The mechanical forces for the directed movement are provided from both tissues and their interface. The squamous amnioserosa cells display pulsatile isotropic contractions which lead to very regular oscillations of the cross-sectional area. On the tissue scale, the oscillations balance out each other due to their asynchrony during the stationary phase preceding dorsal closure. During dorsal closure, however, the contractions take over and lead to a gradual decrease of the total area of the amnioserosa. The decreasing area is compensated or promoted by the movement of the adjacent epidermis. Given several recent excellent reviews on the role of the amnioserosa cells (Hayes and Solon, 2017; Kiehart et al., 2017; Perez-Vale and Peifer, 2020), we will focus on the surrounding epidermis for the closure process in the following paragraphs.

The interface between the two tissues plays an important role. The dorsal-most epidermal cells, the leading edge cells, polarize by an accumulation of F-actin at the interface between their dorsal edge and the amnioserosa interface, which generates a prominent and contractile F-actin cable (Figure 2E) (Young et al., 1993; Kiehart et al., 2000). Meanwhile, the leading edge cells dramatically elongate along the DV direction as if they were pulled by the amnioserosa (Figure 2F) (Jacinto et al., 2002). This notion has remained untested. Both models are conceivable. In the passive model, the elongation of epidermal cells is due to pulling by the amnioserosa cell/actin cable contractions. In the active model, the epidermal cells elongate by an autonomous mechanism within the epidermis and thus generate a pushing force. A combination of both models would also be possible.

Tissue restricted Myo-II depletion in the amnioserosa or surrounding epidermis revealed that the Myo-II dependent contractions within the amnioserosa tissue but not actin cable are required for dorsal closure (Pasakarnis et al., 2016). However, the kinetics of the overall closure process appeared slower when Myo-II was depleted in the epidermis. Myo-II depletion in epidermis affects the contractility of all cells of the epidermis, not only the leading edge cells and the actin cable. Yet unidentified autonomous mechanisms could be affected within the epidermis. It is worth noting that Myo-II depletion specifically within the amnioserosa, also affected the actin cable structure (Pasakarnis et al., 2016). In these embryos, the actin cable initially formed but the cable structure disassembled partially during dorsal closure. These observations suggest a role of amnioserosa cell contractions for the cable structure. The elongation of epidermal cells might be due to pulling by the actin cable tension. The tension along the actin cable increases steadily over time, as revealed by the recoil velocity following UV laser-induced junction cutting (Saias et al., 2015). Opposing a role of the actin cable comes from the analysis of *Zasp52* mutants embryos, which lack any actin cable but undergo an apparently normal dorsal closure (Ducuing and Vincent, 2016). Interestingly, the elongation of epidermal cells is still observed in *Zasp52* mutants. These observations suggest that the elongation of epidermal cells is not only due to pulling by the actin cable.

## Adherens Junctions at the Leading Edge Cells

Although the amnioserosa cells behave isotropically with respect to their oscillations, the cell junctions at the interface are polarized as seen not only by the actin cable but also by the junction and junction-associated proteins. The epidermis connects with amnioserosa cells via E-cad and integrin-mediated adhesions (Narasimha and Brown, 2004). Reduced E-cad levels impair cell contacts between leading edge cells and amnioserosa (Gorfinkiel and Arias, 2007). Correspondingly interface defects within the actin cable and edge cells of the amnioserosa were observed in α*-catenin* mutant embryos, in which the actin-binding domain was specifically deleted (Jurado et al., 2016). Further actin-binding proteins associated with adherens junctions were recently identified to localize at the interface. Although Canoe and Polychaetoid are not essential for the actin cable, the architecture and morphology of leading-edge cells were impaired in embryos depleted for those proteins (Manning et al., 2019). The Ajuba LIM protein (Jub), a force-sensitive protein, is enriched at the interface, and loss of Jub enhances dorsal closure defects in mutants defective for cell adhesion (Razzell et al., 2018). This protein accumulates at adherens junctions under tension and acts as a critical component of a negative-feedback loop, which stabilizes and distributes tension at adherens junctions at the interface (Rauskolb et al., 2019). These studies strongly suggest that adherens junctions have fundamental functions in adapting to mechanical forces and coordinate the tissue and cell interactions leading to morphogenesis.

## Conclusion and Remarks

Within the lateral epidermis during gastrulation, the AP patterning system establishes a system of planar cell polarity, which polarizes junctional and cytoskeletal dynamics and subsequently directs cell rearrangement for the tissue-scale changes in morphology. The finding that members of the Toll-family of membrane receptors are involved in the polarization of the tissue has started to open the black box of molecular links between the transcriptional patterning machinery for axis formation and the cell biological machinery of contractile actomyosin clusters and cell adhesion complexes (Paré et al., 2014; Tetley et al., 2016; Lavalou et al., 2020). E-cad adhesion complexes are at the core of mechanical coordination between neighbors in epithelia. Its potential functions and the interactions with contractile actomyosin networks and other interaction partners provide ample options for fine-tuning sensory and signaling functions.

Yet missing is an integrative systems-type analysis involving mechanisms of coordination among the direct neighbors but also long-ranging influences to second and third neighbors. Analysis of the temporal and spatial coordination of the identified contractile and adhesive activities will be needed for the step from understanding the individual events such as a junction collapse to the tissue-scale shape changes during morphogenesis. *Drosophila* embryos provide a suitable and highly tractable system to study such questions *in vivo*.

Beyond the individual tissue, polarized and anisotropic tensions from the neighboring tissues have a potentially big impact on morphogenetic processes. The anisotropic tension by the posterior midgut during gastrulation pulls on the lateral epidermis, which is visible by a corresponding AP stretching of the cells during the onset of germband extension (Lye et al., 2015). This anisotropic tension with a gradual increase toward the posterior tip of the embryos transiently orientates newly formed junctions (Collinet et al., 2015). During germband extension cell stretching is diminished by cell rearrangement, even though the polarized tension remains on the tissue scale (Collinet et al., 2015; Lye et al., 2015). For a full understanding, it needs to be investigated whether and how E-Cad complexes and its interacting partners are involved in the coordination of local and tissue-scale forces during epithelium morphogenesis.

Similar tissue interactions are essential for the morphogenesis of the amnioserosa and dorsal closure. The two sheets of the dorsal epidermis are exposed to an anisotropic tension from the pulsating and contracting amnioserosa as well as the contractile actin cable. Cell elongation occurs not only in the leading edge cells but also in the further distant second and third and so forth neighbors in the epidermis (Figure 2F). It has remained unclear to which degree the elongation of the epidermal cells contributes to the closure process. How does the dorsal epidermis respond to and coordinate the polarized anisotropic tension with the cell shape changes? Adherens junctions and the binding proteins could be the potential candidates. For example, Arf-GEF Steppke is recruited to the myosin-rich adherens junction via coiled-coil heterodimerization with an adaptor protein, where the complex downregulates junctional tension and facilitates tissue stretching (West et al., 2017; Zheng et al., 2019). It is worth expanding the research focus from the amnioserosa and actin cable to the surrounding epidermis. As stated above the numerous proteins and processes associated with E-cad core complexes provide ample options for regulation and fine-tuning of morphogenetic processes.

## Author Contributions

DK wrote the manuscript and drew the figures. JG revised the manuscript. JG and DK conceived the study and edited the manuscript. Both authors contributed to the article and approved the submitted version.

## Conflict of Interest

The authors declare that the research was conducted in the absence of any commercial or financial relationships that could be construed as a potential conflict of interest.
